# Mixture-Based Combinatorial Libraries from Small Individual Peptide Libraries: A Case Study on α_1_-Antitrypsin Deficiency

**DOI:** 10.3390/molecules19056330

**Published:** 2014-05-16

**Authors:** Yi-Pin Chang, Yen-Ho Chu

**Affiliations:** 1 The Forsyth Institute, 245 First Street, Cambridge, MA 02142, USA; 2 Department of Chemistry and Biochemistry, National Chung Cheng University, 168 University Road, Minhsiung, Chiayi 62102, Taiwan

**Keywords:** antitrypsin, alanine scanning, conformational disease, iterative deconvolution, peptide library, positional scanning, split-and-mix, surface plasmon resonance, truncation library, urea gel

## Abstract

The design, synthesis and screening of diversity-oriented peptide libraries using a “libraries from libraries” strategy for the development of inhibitors of α_1_-antitrypsin deficiency are described. The major buttress of the biochemical approach presented here is the use of well-established solid-phase split-and-mix method for the generation of mixture-based libraries. The combinatorial technique iterative deconvolution was employed for library screening. While molecular diversity is the general consideration of combinatorial libraries, exquisite design through systematic screening of small individual libraries is a prerequisite for effective library screening and can avoid potential problems in some cases. This review will also illustrate how large peptide libraries were designed, as well as how a conformation-sensitive assay was developed based on the mechanism of the conformational disease. Finally, the combinatorially selected peptide inhibitor capable of blocking abnormal protein aggregation will be characterized by biophysical, cellular and computational methods.

## 1. Introduction

Combinatorial chemistry in drug discovery represents an amalgamation of chemistry and biology, which generally involves the practice of chemical synthetic methods and coupled with biological screening assays. It allows rapid preparation and high-throughput screening of compound libraries for the identification of drug candidates or optimization of lead compounds. This technology has been widely and routinely used by researchers in industry and academia since the 1990s, and holds promise to accelerate the protracted time of drug development process. The first combinatorially identified drug (sorafenib) was approved by the U.S. Food and Drug Administration (FDA) for clinical treatment of kidney cancer, liver cancer and thyroid carcinoma in 2005 [1]. In retrospect, the roots of combinatorial chemistry have inseparable relations with the development of solid-phase synthesis. In 1963, Merrifield pioneered solid-phase peptide synthesis (SPPS) and described the preparation of a 4-mer peptide [2]. Since then, combinatorial synthesis emerged in parallel in several groups and their approaches mushroomed almost simultaneously. Leznoff synthesized small molecules on insoluble polymer supports [3,4]. Frank came up with the idea to synthesize nucleotides and later peptides on cellulose papers [5]. Geysen performed solid-phase synthesis on polymeric pins arranged in 96-well microtiter plates [6,7], whereas Houghten carried out peptide synthesis on “tea bags” [8]. Furka devised the concept of split-and-mix method, and it was applied to the solid-phase synthesis of peptide libraries [9]. Lam and co-workers conceived the method to prepare peptide libraries and screening on beads [10], while Houghten used the split-and-mix and tea-bag approaches to make soluble peptide libraries [11]. The endeavor continues and has brought combinatorial technology into many knotty biological problems such as α_1_-antitrypsin deficiency (AATD), a proteopathy or protein conformational disorder.

Peptide libraries are a key research tool for the search of bioactive compounds by screening large numbers (tens of thousands or even billions) of peptides that is not practically attainable by traditional chemical approach. These synthetic libraries can be prepared as either mixtures or sets of individual peptides by using *tert*-butyloxycarbonyl (Boc) or 9-fluorenylmethyloxycarbonyl (Fmoc) SPPS methods. The relative ease of SPPS makes assay development and screening strategy the central parts of peptide library screening, in which its applications including but not limited to epitope mapping of antibodies and substrate profiling of proteases. These established approaches normally utilize either library that contains individual compounds or mixtures for the target of interest, which are feasible when the binding sites or ligand scaffolds are well-defined [12,13,14]. However, if the druggability of a protein is difficult to fathom due to the lack of structural information or in some cases the promiscuity of binding, library screening can be a formidable task and requires a design in depth strategy. Herein we illustrate how a peptide inhibitor of AATD was identified through small focused libraries and a diversity-oriented library.

**Figure 1:**
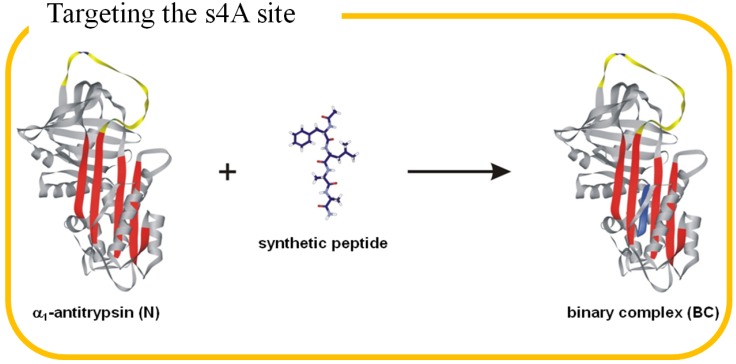
Targeting the s4A site on the β-sheet of α_1_-antitrypsin (AT). The native AT (PDB code: 1QLP) contains a reactive center loop (yellow) and a β-sheet A (red) which are the starting points of the pathogenic polymerization. The five strands of the β-sheet are termed as s1A, s2A, s3A, s5A and s6A (from left to right). The synthetic peptide (blue) inserts into the s4A position to form binary complex (BC) and thus blocks the propagation of polymerization.

AATD is an inherited condition that causes liver cirrhosis and lung emphysema. Because of the unspectacular progression of its symptoms, AATD remains one of the most hidden killers in the world. Laurell and Eriksson first described AATD from young patients with emphysema in Sweden in 1963 [15,16]. Interestingly, this commonly underdiagnosed disorder has been considered to responsible for the premature death of the Polish composer Frédéric François Chopin [17]. The second common manifestation cirrhosis was described afterwards [18]. The World Health Organization (WHO) suggested that the frequency of AATD in Europe and North America is comparable to that of cystic fibrosis, at 1 in 7,000 to 1 in 20,000 [19]. For the past few decades, researchers unraveled the crystal structure (PDB code: 1QLP), inhibitory function, pathogenic mechanism and clinical manifestation of α_1_-antitrypsin (AT), which provided the knowledge for several therapeutic models. Blocking the abnormal aggregation of AT by targeting “s4A” (the 4th strand on the β-sheet) with synthetic peptide is a promising strategy (Figure 1), but the lack of molecular diversity and effective assay preclude the discovery of potent inhibitors. Combinatorial peptide libraries appeared to be an excellent arsenal to cope with this clinical problem. We first screened small libraries (alanine scanning, truncation, D-amino acid scanning and positional scanning libraries) which facilitated the design of a large mixture library and thereby identified the most potent AATD inhibitor to the best of our knowledge. The initial design of the libraries was inspired by the intriguing inhibitory mechanism of AT, in which shared by the serine protease inhibitor (serpin) family. The combinatorial approach will be described in detail.

## 2. α1-Antitrypsin Deficiency (AATD)

### 2.1. Mechanism of AATD-Associated Liver and Lung Diseases

AATD is a protein conformational disorder (also known as protein misfolding disease or simply proteopathy) along with other notorious clinical conditions such as Alzheimer's disease, amyloidosis, Parkinson's disease, prion disease and type 2 diabetes [20]. AT is a major protease inhibitor within the lungs, but is primarily synthesized by the hepatocytes in the liver. This glycoprotein consists of 394 amino acids and serves as a suicide substrate to protect connective tissues in the lungs from destruction by inhibiting its cognate enzyme neutrophil elastase (NE) [21]. Individuals without sufficient circulating levels of AT to neutralize NE predispose lung diseases such as emphysema [22]. A single-nucleotide mutation in the gene (14q32.1) can cause liver deposition and consequently plasma deficiency, which manifests cirrhosis and emphysema, respectively. Among the alleles of I (Arg39Cys), Mmalton (ΔPhe52), S (Gly264Val), Siiyama (Ser53Phe) and Z (Glu342Lys) alleles, the Z variant of AT causes the most significant deficiency (about 90%) compared with the wild-type M allele [23]. It has been estimated that at least 116 million carriers are with the mild combination alleles of MS and MZ, while 3.4 million individuals are with the SS, SZ and ZZ severe alleles worldwide [24]. The molecular basis of AATD lies in the structural rearrangement of the mutant proteins and is the key to develop therapeutic approaches.

The disease mechanism of AATD has a close connection with the mobility nature of inhibitory serpins. The tertiary structure of serpins shares a conserved core domain, which includes the three dominant β-sheets and about nine α-helices [25]. During the native state of the inhibitory AT, a unique 17-residue reactive center loop (RCL) is exposed to entrap its targeted protease NE. Following the cleavage of the scissile bond by NE, the RCL swings and incorporates itself into the β-sheet. This event concomitantly translocates NE more than 70 Å to the opposite end and forms a 1:1 enzyme:inhibitor complex, which renders the β-sheet fully antiparallel [26]. Unlike most of the other classes of protein protease inhibitors that use a lock-and-key fashion to form tight but reversible noncovalent complex between proteases and their corresponding inhibitors, inhibitory serpins involve the two-stage inhibitory mechanism: (1) rapid formation of a noncovalent Michaelis complex, attack of the scissile bond to form a tetrahedral intermediate and subsequent cleavage to give the covalent acyl-enzyme intermediate; and (2) insertion of the cleaved RCL into the β-sheet and stabilization of the second tetrahedral intermediate through the attack of water [27]. The dynamic features of the serpin inhibitory mechanism ensure the suicidal and irreversible inhibition, in which the RCL plays a pivotal role, or, more precisely, a double-edged sword.

### 2.2. Loop-Sheet Polymerization of Serpins

**Figure 2:**
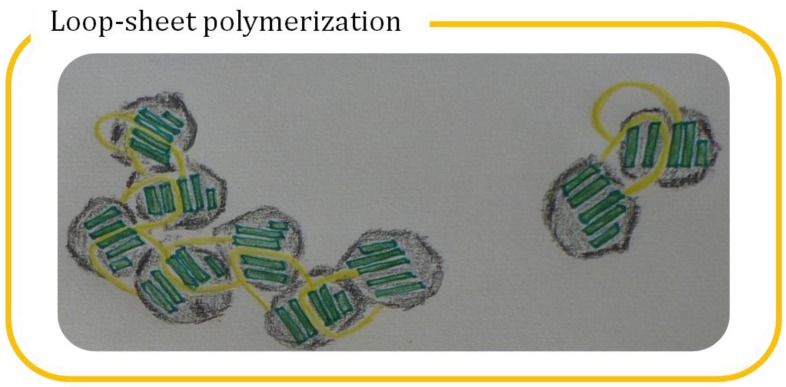
Loop-sheet polymerization of α_1_-antitrypsin (AT). This abstract sketch illustrates how a dimer and an oligomer of several AT molecules are linked through their reactive center loop (yellow) and β-sheet (green). Drawing courtesy of Ms. Lucie Drdova.

Abnormal protein aggregation is the culprit of AATD. The pathogenic aggregates are derived from the intermolecular linkages of mutated AT, in which the spontaneous phenomenon is coined as “loop-sheet polymerization” [28]. In spite of the crucial role of RCL in its inhibitory function, point mutations of AT perturb the protein structure and render the β-sheet susceptible to be inserted by an exogenous RCL of an adjacent AT to form a dimer. Furthermore, the exposed RCL and unoccupied β-sheet on the newly-formed dimer are available for more linkages to propagate oligomers and eventually polymers (Figure 2). For example, Z mutation disrupts the salt bridge (Glu342–Lys290) of AT and the substitution of the hinge residue not only extends the RCL but also expands the β-sheet as a donor and receptor for the propagation of polymerization, respectively [29]. Intracellular polymers of Mmalton, Siiyama and Z alleles have been identified within the endoplasmic reticulum (ER) of hepatocytes, where AT is synthesized and cause chronic liver injuries. The ER retention of polymeric AT causes the secretory defection and results in plasma deficiency, which adversely makes the lungs vulnerable to elastolytic damage.

In view of the conserved structure of serpins, it is not surprising that the same mechanism of disease have been identified among serpinopathies, such as α_1_-antichymotrypsin (ACT; Leu55Pro and Pro228Ala), antithrombin (ATIII; Pro54Thr and Asn158Asp), C1 inhibitor (Phe52Ser, Pro54Leu, Ala349Thr, Val366Met, Phe370Ser and Pro391Ser) and neuroserpin (Ser49Pro) in association with emphysema, thrombosis, angio-oedema and dementia, respectively. Crystallographic structures of serpins support the hypothesis of loop-sheet polymerization and provide insights into the propagation of pathogenic polymers. While loop-sheet polymerization remains a classic model for serpinopathies, a recent crystal structure of a stable antithrombin dimer provides a plausible elucidation for serpin polymerization [30]. Rather than the insertion of a single-chain RCL, a RCL together with its adjacent strand participate the “β-hairpin domain swapping” to form the dimerized structure. Taken together, RCL is all in all the key component of the pathogenic polymerization, in which the molecular basis offers a therapeutic strategy for AATD-associated liver and lung diseases.

## 3. The Peptide Annealing Method

### 3.1. RCL Peptides Bind to Serpins

The peptide annealing method is basically an “eye for an eye” strategy. Given that the inhibitory mechanism and pathogenic polymerization of serpins are both conducted by the incorporation of RCL, it appears that RCL peptides can compete with endogenous RCL at the same binding site and thus intervene in the propagation of polymerization. In retrospect, RCL peptides raging from 8-mer to 16-mer of AT, ATIII and PAI-1 were synthesized to probe the structural transition, inhibitory activity, conformational stability, polymerization mechanism and physical properties of serpins in the 1990s [31,32,33,34,35,36,37,38,39,40,41,42,43,44]. These RCL peptides answered some long-sought-after puzzles of serpins, but were weak and nonspecific binders. They typically required high concentrations (100–200 fold molar excess) and days of incubation to form binary complex. In addition, the binding was promiscuous such as a RCL peptide of ATIII could bind to ATIII, ACT and AT [42,44,45,46]. Spectroscopic techniques (circular dichroism and tryptophan fluorescence), polyacrylamide gel electrophoresis (native- and SDS-PAGE) and antitryptic activity assessment (residual tryptic activity) were used for the detection of binary complex by monitoring the changes of molecular weight, conformational change and enzymatic activity, respectively. These early studies represent the proof-of-concept of the peptide annealing method.

A short 6-mer RCL peptide (Ac-FLEAIG-OH) was used to explore the structural differences between the pathogenic Z-AT and normal M-AT [47]. Despite that the sequence was derived from the RCL of AT, the peptide preferentially annealed to Z-AT and did not significantly bind to other serpins (ATIII, ACT and PAI-1) that bear the similar tertiary structure. The assessment of peptide binding was achieved by intrinsic tryptophan fluorescence and native-PAGE as previous works. In addition, the adulterated urea increased the readout of native-PAGE, which distinguished the binary complex from unbound AT unambiguously. The effect of the chaotropic reagent was imperative for this conformation-sensitive gel electrophoresis to generate distinct band shifts. To further unravel how small peptides interact with serpins, a structural study assessed the binding of around 40 RCL and exogenous peptides [46]. Threonine on the peptides seemed to facilitate the entry and anchoring of peptide annealing into the β-sheet, which correlated with a separate study on the role of threonine as a key stabilizer in the hydrogen bond network that plays a crucial role in maintaining the metastable conformation of serpins [48]. The crystal structures of the ATIII ternary complexes also demonstrated the importance of hydrophobic side chains on the peptides. Taken together, these pioneered works provide prototypes for assay development and some structural information for the design of combinatorial libraries.

### 3.2. Small Individual Peptide Libraries

Research focused on understanding the underlying characteristics of a disease can often lead to promising strategies for drug development. Considering “loop-sheet polymerization” is the disease mechanism of AATD and the fact that RCL peptides bind to serpins, targeting the s4A site with peptide libraries is therefore intriguing [49]. Despite this straightforward concept, the development of a combinatorial approach for the conformational disease AATD requires detailed insights for the design of large libraries. In addition, the lack of appropriate high-throughput screening platform was one of the main obstacles in this area. To this end, a pilot study using small individual peptide libraries was conducted to define structural requirements and minimal peptide length for effective binding, as well as to develop an assay for iterative deconvolution.

**Figure 3:**
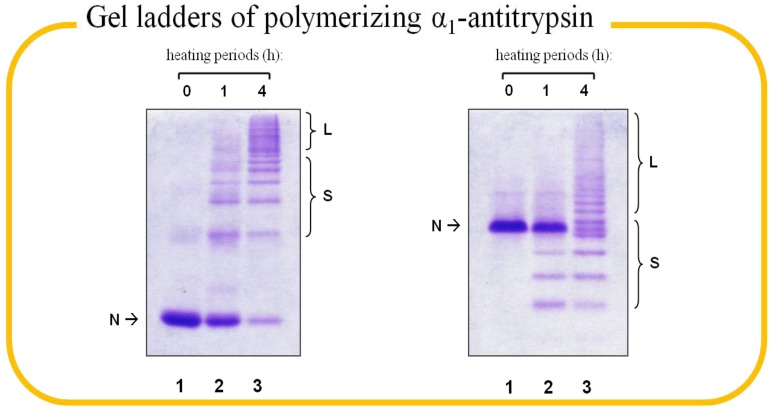
Gel patterns of polymerizing α_1_-antitrypsin (AT). 8% (*w/v*) native-PAGE (left) and 8% (*w/v*) 8 M urea native-PAGE (right) demonstrating the patterns of heat-induced polymerization of M-AT (4 μg, heated at 58 °C) at 0 (lane 1), 1 (lane 2), and 4 (lane 3) h. The native protein (N), short-chain (S), and long chain (L) polymers of AT are marked in the figure.

**Figure 4:**
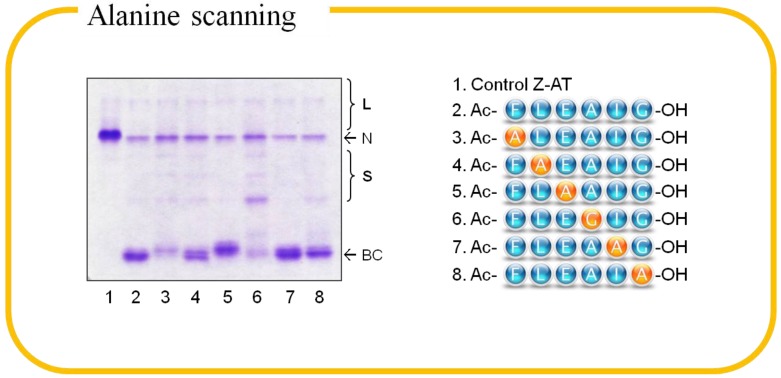
8% (*w/v*) 8 M urea native-PAGE demonstrating the result of alanine scanning of the peptide Ac-FLEAIG-OH. Z α_1_-antitrypsin (N) was incubated with a 100-fold molar excess of the peptides at 37 °C for 3 days. Peptides capable of binding to the protein exhibit clear binary complexes (BC) and lesser ladder bands of the protein oligomers on the gel.

The peptide (Ac-FLEAIG-OH) corresponds to the P7-P2 segment of AT RCL was selected as the starting point for the combinatorial approach. First, this benchmark peptide binds to Z-AT and blocks its polymerization. Second, this sequence is likely to play a pivotal role in both binding affinity and specificity for each protease inhibitor system, as P7-P2 is the only region that is not conserved among RCL of inhibitory serpins [27]. We first prepared an alanine scanning library to dissect the binding interface of AT and to unveil the contribution of each residue to peptide annealing [50]. Alanine scanning is a technique that has been extensively used to identify critical residues for protein function, stability and conformation in molecular biology [51]. The smallest chiral amino acid alanine was used to substitute each non-alanine residue based on the 6-mer peptide one at a time to construct the small individual peptide library for screening. Initially, the *in vitro* library screening was based on conventional PAGE, however, unbound protein and binary complex could not be distinguished unambiguously if the binding peptide had no charged amino acid. In conjunction with heat-induced polymerization, we were able to identify the binding peptides by native-PAGE, but the harsh condition might disrupt the native state of protein and produce artefacts. Eventually, we solved the issues by adulterating urea in native-PAGE, which generated distinct band shifts and thus greatly improved the readout. The patterns of how AT behaves in the two gel-based assays are shown in Figure 3. The screening of alanine scanning library identified two 6-mer peptides (E3A: Ac-FLAAIG-OH and I5A: Ac-FLEAAG-OH) that bound to M- and Z-AT (Figure 4). Hydrophobic residues appeared to be significant at the corresponding P6 (leucine and alanine) and P4 (alanine) sites, which correlated with the crystal structure of ATIII complexes [46]. On the other hand, glycine was deleterious for binding, in which the achiral amino acid was likely to make the conformation of the peptide not compatible at the binding interface.

**Figure 5:**
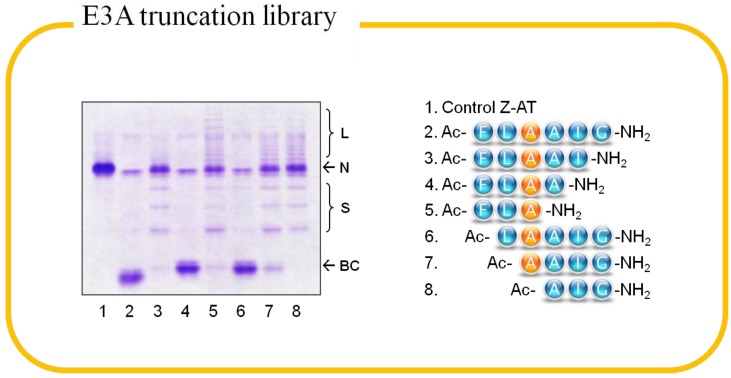
8% (*w/v*) 8 M urea native-PAGE demonstrating the result of truncation library of the peptide Ac-FLAAIG-OH (E3A). Z α_1_-antitrypsin (N) was incubated with a 100-fold molarexcess of the peptides at 37 °C for 3 days. Peptides capable of binding to the protein exhibit clear binary complexes (BC) and lesser ladder bands of the protein oligomers on the gel.

Truncation libraries of the two 6-mer peptides identified from alanine scanning were prepared to determine the minimum peptide length required for binding [52]. The incremental truncation of the two peptides (E3A and I5A) from both N- and C-terminal ends generated 14 individual peptides, which lead to the identification of two daughter peptides Ac-FLEAA-NH_2_ and Ac-FLAA-NH_2_ that bound to Z-AT avidly and inhibited the polymerization [50]. Therefore, a 4-mer peptide was sufficient for peptide annealing. A representative result of E3A truncation library is shown in Figure 5. Next, we examined the effect of N-terminal acetylation and C-terminal amidation of the 4-mer peptide Ac-FLAA-NH_2_ and its three analogs (FLAA-NH_2_, Ac-FLAA-OH and FLAA-OH). Although the 4 analogs bear the same peptide sequence all bound to AT, their affinity varied with the N- and C-terminal modifications. The N-terminal acetylated peptides bound to AT more tightly than their corresponding N-terminal free peptides, perhaps due to the extra hydrogen bond acceptor on the acetyl group. The peptide amides exhibited higher affinity than their peptide acid counterparts, which might, because of the elimination of the negative charge. Taken together, N-terminal acetylated 4-mer peptide amide was the best candidate for the design of mixture-based libraries. We expected this kind of peptides would have better stability against aminopeptidases and synthetases, as well as increased cell permeability. The effect of D-amino acids on the 4-mer peptide Ac-FLAA-NH_2_ was evaluated, but most of the D-substituted peptides were not able to form binary complex with Z-AT indicating a stereochemical constraint of the binding interface [50].

### 3.3. Mixture-Based Combinatorial Libraries

Through the systematic screening of small individual peptide libraries, the scaffold for the mixture-based peptide libraries was defined. The next step was the selection of building blocks for the chemical synthesis of libraries. In principle, a peptide library can attain the maximum molecular diversity if all the 20 proteinogenic amino acids are incorporated. It is an aesthetic goal to synthesize a fully combinatorial library consists of all the possible peptide sequences, but some practical issues may arise in some cases if the library was not design judiciously. Based on the previous screening conditions, a conservative estimate indicated a potential solubility issue may occur if the library size was not well controlled. As a result, a selection criterion of building blocks was needed to avoid futile screening.

**Figure 6:**
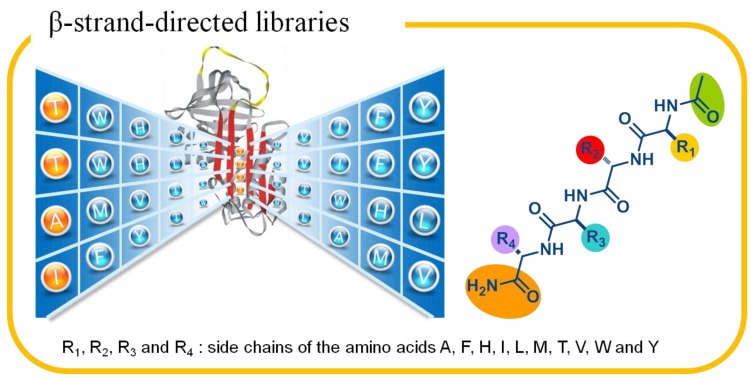
Targeting the s4A site with β-strand-directed libraries. The 10 β-sheet preferred amino acids (A, F, H, I, L, M, T, V, W and Y) are selected for the building blocks of the acetylated tetrapeptide library to probe the s4A site of α_1_-antitrypsin.

Insights into the inhibitory mechanism of serpins provided an excellent criterion for the selection of building blocks. In light of the inhibitory mechanism, RCLs are flexible and can adopt a number of diverse conformations, but upon snaring their cognate enzymes, each RCL inserts into their corresponding β-sheet and render it a fully anti-parallel β-sheet conformation. Therefore, the residues within RCL of inhibitory serpins must be compatible with the β-sheet to complete the inhibitory mechanism, which are most likely the β-sheet preferred amino acids. To confirm this hypothesis, the RCL sequence of 34 human serpins was analyzed in conjunction with the Chou–Fasman helix and sheet propensities in the prediction of secondary structure [53]. According to the analysis, it may not be a coincidence that 73% of the RCL residues of inhibitory serpins were the top 10 β-sheet preferred amino acids (A, F, H, I, L, M, T, V, W and Y). On the contrary, only 38% of the amino acids were found in the RCL of non-inhibitory serpins. The diverse preference of amino acids clearly supported the hypothesis and thus the 10 amino acids were selected for the construction of the mixture-based libraries which was named as β-strand-directed libraries (Figure 6). Interestingly, the 10 selected amino acids not only include the previously identified alanine and leucine from the alanine scanning library, but also exclude glycine.

Iterative deconvolution was employed to stepwise determine the optimal residues for M- and Z-AT binding by cycles of synthesis and screening of libraries. A virtue of this iterative method is the enrichment process, in which the diversity of components decreases upon each step of screening and therefore active individual hits can be identified from active pool of mixtures eventually [54]. It essentially involves a set of mixture sub-libraries with defined positions, which are synthesized by the solid-phase split-and-mix method [9]. The first generation of β-strand-directed libraries (Ac-X_1_X_2_X_3_X_4_-NH_2_) were constructed based on the scaffold of N-terminal acetylated 4-mer peptide amide and thus 4 cycles of synthesis and screening were exhaustively repeated to identify the active sequences. The size of libraries decreased exponentially until all the residues were defined in the final individual library (from 10,000 to 1,000 and then 100 peptide mixtures, and finally 10 individual peptides). The chemical synthesis of libraries initiated at the X_4_ position and completed at the X_1_ position, so the elongation of peptide chains were from C-terminal to N-terminal. Upon completion of the 4th reaction cycle (coupling of the amino acids at the X_1_ position), the generated 10 sub-libraries (each contains 1,000 peptides) were not mixed and therefore the N-terminal amino acids (X_1_) were known, which were designated as Ac-AX_2_X_3_X_4_-NH_2_, Ac-FX_2_X_3_X_4_-NH_2_, Ac-HX_2_X_3_X_4_-NH_2_, Ac-IX_2_X_3_X_4_-NH_2_, Ac-LX_2_X_3_X_4_-NH_2_, Ac-MX_2_X_3_X_4_-NH_2_, Ac-TX_2_X_3_X_4_-NH_2_, Ac-VX_2_X_3_X_4_-NH_2_, Ac-WX_2_X_3_X_4_-NH_2_ and Ac-YX_2_X_3_X_4_-NH_2_. Each randomized position (Xi) was a combination of the 10 selected amino acids.

**Figure 7:**
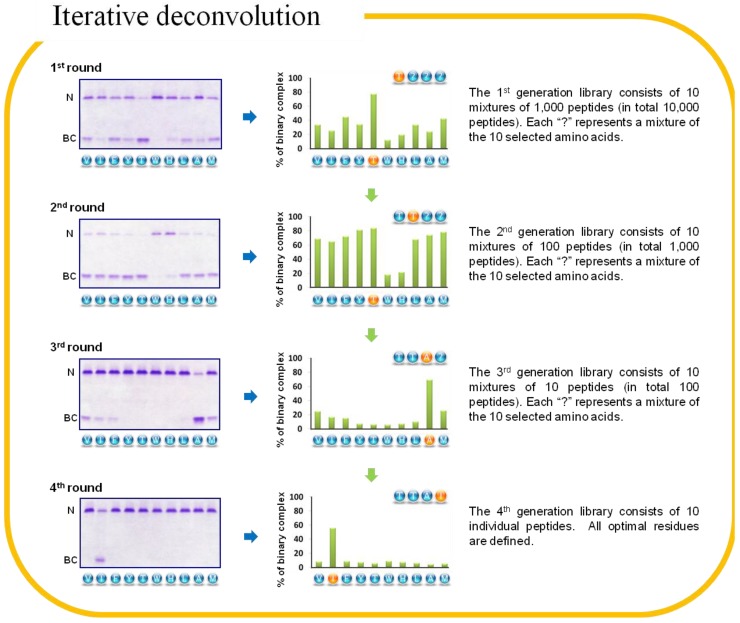
8% (*w/v*) 8 M urea native-PAGE demonstrating the result of iterative deconvolution of Z α_1_-antitrypsin (AT). The potency of each sub-library was determined by percentage of binary complex (BC) formation, which was quantitatively measured by densitometric analysis. The most reactive sub-library in each screening cycle is indicated on the top right corner of each bar graph. All screenings were performed with a 10-fold molar excess of each individual peptide against Z-AT at 37 °C for 2 h except the last round was only 1 h.

The primary assay for deconvolution was a conformation-sensitive native-PAGE containing urea [50]. As shown in Figure 7, the first three cycles of screening identified the residues of T, T and A from the libraries of Ac-X_1_X_2_X_3_X_4_-NH_2_, Ac-TX_2_X_3_X_4_-NH_2_ and Ac-TTX_3_X_4_-NH_2_, respectively. In the last cycle of screening, the optimal Z-AT-binding peptide was revealed as Ac-TTAI-NH_2_ from the final individual library of Ac-TTAX_4_-NH_2_. The potent binding affinity and specificity of the combinatorially selected peptide were demonstrated as it only required 10-fold molar excess and 1 h of incubation to form clear binary complex [55]. Under the same stringent condition, the binary complex of M-AT and Ac-TTAI-NH_2_ was hardly seen. The optimal M-AT-binding peptide was also identified, a very close sequence, Ac-TTAF-NH_2_.

### 3.4. Positional Scanning Library

Positional scanning libraries are useful tools for peptide sequence optimization [56]. There are several types of the library but basically it screens amino acid of interest at given position. This technique was used to validate the screening result of the β-strand-directed library. A potential issue of the split-and-mix method is that the most potent compound may not be identified due to synergistic effect. This effect should be taken into account particularly if the potency of each sub-library could not be distinguished unambiguously. In retrospect, the most potent residue at the X_2_ position of the β-strand-directed libraries was not able to distinguish without the precise densitometric analysis. Although threonine was selected for the synthesis of Ac-TTX_3_X_4_-NH_2_ and identified potent M- and Z-AT binders (Ac-TTAF-NH_2_ and Ac-TTAI-NH_2_, respectively), we suspected if there was a synergy of various peptides bearing the sequence Ac-TTX_3_X_4_-NH_2_. To this end, a positional scanning library (Ac-TX_2_AF-NH_2_; X_2_ = A, H, I, L, M, F, T, V, W and Y) was prepared and screened against M-AT. This small individual library can be taken as part of the β-strand-directed library Ac-TX_2_X_3_X_4_-NH_2_ (a mixture of 1,000 peptides). The positional scanning-retrieved peptides Ac-TTAF-NH_2_ along with other Ac-TX_2_AF-NH_2_ peptides other than Ac-TWAF-NH_2_ were found to bind to M-AT (see Figure S1 in Supplementary Material), which indicated the tolerance at the X_2_ position. It requires the synthesis of all the 100 individual peptides of Ac-TTX_3_X_4_-NH_2_ to essentially rule out synergistic effect, which may not be practical, as the residues at X_1_, X_3_ and X_4_ were clearly defined. It appears that synergy is probably inevitable in most mixture-based peptide libraries. The inherent nature may or may not facilitate the discovery of potent compounds. It depends on the selection of building blocks that all goes back to the initial design of libraries.

## 4. Characterization of the Combinatorally Selected Peptide

### 4.1. Surface Plasmon Resonance

**Figure 8:**
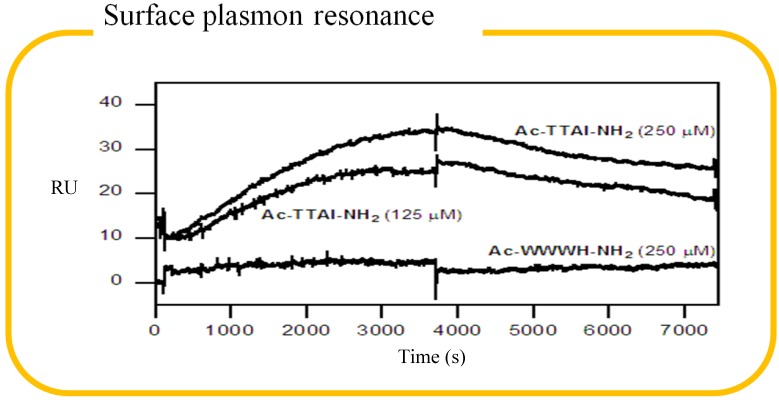
Surface plasmon resonance showing the binding of the combinatorially selected peptide to a Z α_1_-antitrypsin (AT) chip. Overlay plot demonstrating the specific binding of Ac-TTAI-NH_2_ (125 and 250 μM), as the control Ac-WWWH-NH_2_ (250 μM) is not recognizedby the immobilized Z-AT. The sensorgrams of Ac-TTAI-NH_2_ were shifted (10 RU) for clarity.

Ever since AATD was first described in 1963, researchers have been fascinated by the molecular events related to the serpin superfamily. It has been a mystery on how RCL peptides bind to serpins, and we were curious about the binding kinetics of the combinatorally selected peptide. The main question was why it took hours of incubation, but once the binary complex was formed, it seemed hardly fell apart? In virtue of the end-point gel assay was not able to answer the question due to its discontinuous nature. As a result, the peptide Ac-TTAI-NH_2_ was further assessed by surface plasmon resonance (SPR) to dissect the binding events to Z-AT. In addition, this chip-based biosensor is a powerful tool for biomolecular interaction analysis and can serve as an excellent platform to validate the gel-based library screening. The monitoring of protein-peptide interaction by SPR is real-time and without the need of tags or labels [57,58]. We intentionally immobilized Z-AT onto the sensor chip to avoid any hindrance of protein-peptide interactions that might result from the immobilization chemistry. The preliminary sensorgrams showed that the binding of Ac-TTAI-NH_2_ to the immobilized Z-AT was specific and in a dose-dependent manner (Figure 8). The negative control Ac-WWWH-NH_2_ was hardly recognized by the immobilized Z-AT even at a high concentration. In addition, the slow association rate is in line with the incubation time required to form binary complex, while the slow dissociation rate explains the tight binding, which correlates with the gel-based assay [55]. However, the tight binding of the peptide makes the regeneration condition of sensorchip difficult to optimize, which precludes the measurement of actual *K*_D_ values.

### 4.2. Effects on Cells

The effects of the 4-mer peptide on cells were investigated. The cytotoxicity of Ac-TTAI-NH_2_ was determined by MTT assays against two normal lung epithelial cell lines (BEAS-2B and NL20), two normal lung fibroblast cell lines (WI-38 and IMR-90) and one cancer cell line (A2058). The viability of cells was not affected by the peptide even at a high concentration of 10 μM [55].

Cell models of M- and Z-AT were developed to evaluate the cellular effect on targeting the s4A site as a strategy to inhibit polymerization and restore plasma secretion by synthetic peptides [59]. Among the tested peptides, the combinatorially selected peptide Ac-TTAI-NH_2_ exhibited the most potent activity and not only blocked Z-AT polymerization, but also reversed the pathogenic aggregation and thus improved NE inhibitory capacity and secreted AT concentrations in cells. In addition, the peptide had no adverse effects on apoptosis or cell viability. On the other hand, as Z-AT accumulates in the ER and generates stress that can activate the ER overload response (EOR), the effect of Ac-TTAI-NH_2_ on EOR was assessed [60]. The induction of PERK (protein kinase RNA-like ER kinase)-dependent NF-jB, IL-6, IL-8, and RGS16 and calnexin were abrogated effectively by the peptide, which means the ER stress response due to Z-AT aggregation was also alleviated. Taken together, these results suggest that the peptide is highly effective in blocking the pathogenic cellular activation associated with Z-AT aggregation and ER stress.

### 4.3. Molecular Model

**Figure 9:**
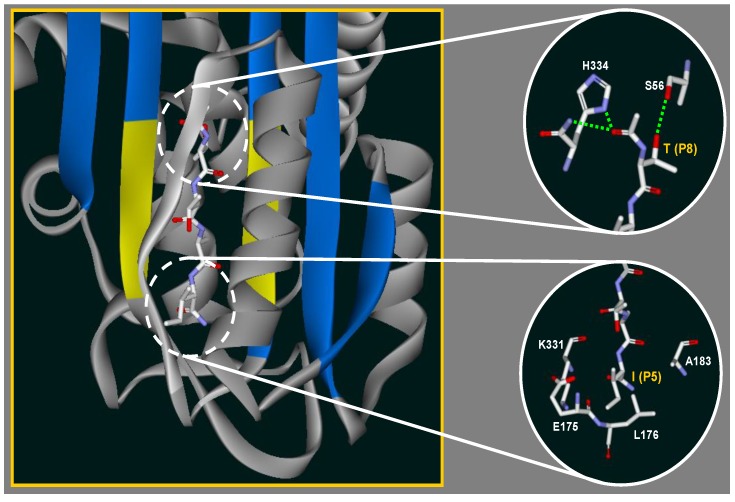
Proposed structure of Ac-TTAI-NH_2_ binding to α_1_-antitrypsin (AT). The peptide inserts into the β-sheet (labeled in blue) of AT and interacts with its nearby residues. The NH and CO groups from the backbone of incorporated peptide are hydrogen bonded to the backbones of adjacent strands (resides in yellow) of AT and render the β-sheet into a 6-stranded antiparallel β-sheet. The circle panel on the upper right reveals the hydrogen bond (light green dashed line) between the N-terminal theronine of Ac-TTAI-NH_2_ and Ser56. Additional hydrogen bonds derive from the acetyl group of peptide with His334 (light green dashed line) and its backbone NH group (light green dashed line) are also illustrated. The other circle panel on the lower right shows the hydrophobic side chain of isoleucine occupies a pocket surrounding by residues K331, E175, L176 and Ala183. Carbon, nitrogen and oxygen atoms are shown in white, light blue and red, respectively.

A structure of the binary complex was proposed to elucidate the possible binding interactions between the identified peptide and the s4A site of AT [55]. The peptide Ac-TTAI-NH_2_ is likely resides at the lower part of the s4A site and lined up with the residues (Ala183, Leu184, Val185 and Asn186) on its right strand and residues (Lys331, Ala332, Val333 and His334) on its left strand (Figure 9). The N-terminal threonine and its acetyl group on the peptide with the side chain of His334, the backbone NH of Lys335 and the side chain of Ser56 form additional hydrogen bonds, respectively. The N-terminal threonine may act as the “anchoring residue” to locate the peptide and initiate the first binding interaction. Next the flexible peptide chain may pinpoint and bind to the β-sheet through backbone hydrogen bonds. Given that the first 3 residues on the peptide all bear small side chains, it is logical that the peptide can evade the connecting loop of the F-helix and incorporate into the β-sheet [61]. Finally, the bulky side chain of the C-terminal isoleucine may occupy the cavity surrounded by Val173, Leu176, Ala183, Glu175 and Lys331, which correlates with a recent report on druggable sites of AT [62]. Taken together, the tight binding of the binary complex is stabilized by hydrogen bonds, hydrophobic interactions and cavity-filling effect.

## 5. Conclusions

The biochemical approach described herein represents a practical example of how chemistry and biology are complementary to shed light on an underdiagnosed clinical problem. *In vivo* data reveal that the combinatorially selected peptide has the potent ability to prevent and reverse intracellular protein aggregation, and to significantly restore secreted functional protease inhibitor. In addition, the identified peptide is able to block pathogenic polymerization and to prevent the cellular consequences of protein accumulation, which demonstrates a real prospect to ameliorate both liver and lung damages in the most severe homozygotes. Moreover, the identified peptide was also used to support the classical loop-sheet polymerization model of serpins [63]. The effectiveness of the “mixture-based libraries from small individual libraries” strategy was demonstrated by the evolution of the initial alanine scanning technique to the truncation, D-amino acid scanning and eventually the β-strand-directed library and its related positional scanning library. These libraries complement to each other and the optimal peptide scaffold was defined for the design of mixture-based library. The developed combinatorial approach should be applicable to other serpinopathies and conformational diseases. 

## Supplementary Materials

Supplementary materials can be accessed at: http://www.mdpi.com/1420-3049/19/5/6330/s1.
