# Supplementary material for: Mixture-Based Combinatorial Libraries from Small Individual Peptide Libraries: A Case Study on α1-Antitrypsin Deficiency
Source: Molecules. 2014 May 16;19(5):6330–48. doi: 10.3390/molecules19056330 (PMC6271437; doi:10.3390/molecules19056330)

Supplementary Material

Figure S1. 8% (*w/v*) 8 M urea native-PAGE demonstrating the result of the positional scanning library (Ac-TX_2AF-NH_2; X_2 = A, H, I, L, M, F, T, V, W and Y). The screening was performed with a 100-fold molar excess of each individual peptide (lane 1 to 10: Ac-TVAF-NH_2, Ac-TIAF-NH_2, Ac-TFAF-NH_2, Ac-TYAF-NH_2, Ac-TTAF-NH_2, Ac-TWAF-NH_2, Ac-THAF-NH_2, Ac-TLAF-NH_2, Ac-TAAF-NH_2 and Ac-TMAF-NH_2) against M-AT at 37 ℃ for 2 h. The peptide Ac-TTAF-NH_2 annealed to M-AT under the same condition as Figure 1A in reference 55, indicating the peptide itself was sufficient to form binary complex. Note there were other 99 peptides in the sub-library of Ac-TTX_3X_4-NH_2 that may or may not bind to M-AT. In addition, the result also showed that a greater tolerance of amino acid at the X_2 position, as 7 out of the 10 selected amino acids were able to form intense binary complex.

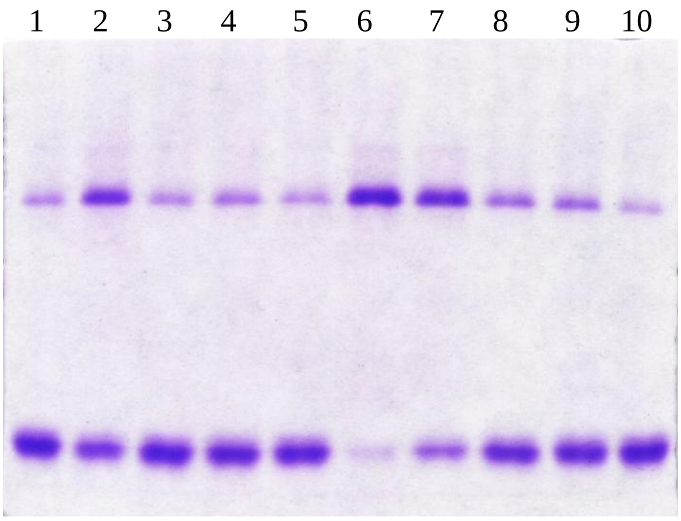

Supplement: Supplementary file 1 [file molecules-19-06330-s001.pdf]
